# Digital psychological self-care for problematic alcohol use: feasibility of a new clinical concept

**DOI:** 10.1192/bjo.2023.73

**Published:** 2023-05-24

**Authors:** Martin Kraepelien, Christopher Sundström, Magnus Johansson, Ekaterina Ivanova

**Affiliations:** Centre for Psychiatry Research, Department of Clinical Neuroscience, Karolinska Institutet & Stockholm Health Care Services, Stockholm, Sweden; and Division of Psychology, Department of Clinical Neuroscience, Karolinska Institutet, Stockholm, Sweden; Centre for Psychiatry Research, Department of Clinical Neuroscience, Karolinska Institutet & Stockholm Health Care Services, Stockholm, Sweden; and Department of Psychology, Stockholm University, Stockholm, Sweden; Centre for Psychiatry Research, Department of Clinical Neuroscience, Karolinska Institutet & Stockholm Health Care Services, Stockholm, Sweden

**Keywords:** Alcohol disorders, cognitive behavioural therapies, digital, intervention, feasibility study

## Abstract

**Background:**

Digital interventions based on cognitive–behavioural therapy and relapse prevention can increase treatment access for people with problematic alcohol use, but for these interventions to be cost-effective, clinician workload needs to remain low while ensuring patient adherence and effects. Digital psychological self-care is the provision of a self-guided digital intervention within a structured care process.

**Aims:**

To investigate the feasibility and preliminary effects of digital psychological self-care for reducing alcohol consumption.

**Method:**

Thirty-six adults with problematic alcohol use received digital psychological self-care during 8 weeks, including telephone assessments as well as filling out self-rated questionnaires, before, directly after and 3 months after the intervention. Intervention adherence, usefulness, credibility and use of clinician time were assessed, along with preliminary effects on alcohol consumption. The study was prospectively registered as a clinical trial (NCT05037630).

**Results:**

Most participants used the intervention daily or several times a week. The digital intervention was regarded as credible and useful, and there were no reported adverse effects. Around 1 h of clinician time per participant was spent on telephone assessments. At the 3-month follow-up, preliminary within-group effects on alcohol consumption were moderate (standardised drinks per week, Hedge's *g* = 0.70, 95% CI = 0.19–1.21; heavy drinking days, Hedge's *g* = 0.60, 95% CI = 0.09–1.11), reflecting a decrease from 23 to 13 drinks per week on average.

**Conclusions:**

Digital psychological self-care for reducing alcohol consumption appears both feasible and preliminarily effective and should be further optimised and studied in larger trials.

Problematic alcohol use causes a considerable part of the global burden of disease.^1^ Despite high prevalence, treatment use continues to be low, with only one in six seeking treatment.^2–4^ In order to attract those reluctant to seek care, it is important to improve accessibility to various forms of addiction services, for example, through the implementation of digital interventions. There has now been more than two decades of research on digital interventions (i.e. internet interventions), demonstrating that they are efficacious for a range of problem areas including depression, anxiety, social phobia and alcohol problems.^5,6^ Over the past decade, these interventions have begun to be implemented within routine healthcare,^7^ a process that the COVID-19 pandemic has probably accelerated.^8^ A recurrent finding of research trials is that digital interventions are more effective when delivered with guidance from a therapist or clinician.^6,9^ However, guidance comes at a cost, as it by definition requires clinician time.^10^ When implementing digital interventions in routine care, clinics may wonder to what extent guidance from a clinician is worth this cost, or whether the added effects of guidance seen in research trials can be achieved in some other more cost-effective way. Indeed, some studies on digital interventions for depression and anxiety suggest that for some participants, there is no consistent difference in effects between self-guided and therapist-guided ones, at least not when the self-guided intervention is delivered within a structured care process.^11^ This care process may entail interviews with clinicians before and after the intervention, clinical monitoring during the intervention to identify deterioration and suicidal ideation, well-designed programmes and weekly standardised automatic messages.^11,12^ Such a structured care process could potentially boost effects of self-guided digital interventions for problematic alcohol use. To illustrate, in two separate trials, the same digital intervention (eChange) was tested in unguided format and compared with a guided intervention. In the first of these trials, where all participants received immediate access to the intervention, adherence and effects in the unguided version were much lower than in the guided version, with participants completing only 21% of the programme on average.^13^ In the second trial, where all participants needed to participate in an initial telephone assessment, fill out weekly measures and participate in follow-up interviews, both adherence and effects in the unguided version were similar to those of the guided intervention, with participants now completing as much as 66% of the programme.^14^ Delivering a self-guided digital intervention in a structured process may thus decrease or even eliminate the difference in adherence and effects between therapist-guided and self-guided digital interventions, while keeping costs related to clinician time lower. The concept of offering a self-guided digital intervention within a structured care process is hereafter referred to as digital psychological self-care (DPSC).

## Aims

The aims of the present study were to evaluate the feasibility and preliminary effects of DPSC for problematic alcohol use. Feasibility measures included intervention adherence, perceived treatment credibility, system usefulness, adverse effects and the use of clinician time. Preliminary effects were assessed with respect to alcohol consumption, alcohol-related problems, alcohol craving and quality of life, as well as depression and anxiety symptoms, up to 3 months after the intervention.

## Method

### Setting

The study was conducted at the Centre for Psychiatry Research, Stockholm Health Care Services, and Karolinska Institutet, Sweden, in cooperation with the digital addiction unit eStöd within the Stockholm Centre for Dependency Disorders, Sweden. The authors assert that all procedures contributing to this work comply with the ethical standards of the relevant national and institutional committees on human experimentation and with the Helsinki Declaration of 1975, as revised in 2008. All procedures involving human subjects/patients were approved by the Swedish Ethical Review Authority (2021-01834), and prospectively registered at Clinicaltrials.gov (NCT05037630).

### Participants and procedure

Participants were recruited by means of advertising on social media platforms and through a national Swedish public support site for individuals with alcohol misuse (www.alkoholhjälpen.se). The recruitment was conducted from 6 September to 25 October 2021. Prospective participants logged into a secure website where they could register interest and provide digital informed consent and contact information, as well as filling out online questionnaires (screening assessment).

To be included participants had to:

- be ≥ 18 years of age;
- have regular access to the internet;
- score ≥8 (for men) or ≥6 (for women) on the Alcohol Use Disorders Identification Test (AUDIT).^15^

Exclusion criteria were:

- insufficient knowledge of the Swedish language;
- difficulties reading or writing related to the use of the digital intervention;
- other ongoing psychological treatment with content similar to that in the current study (problematic alcohol use);
- high suicide risk based on telephone assessment;
- other urgent need for more intensive psychiatric care, or addiction care services, based on telephone assessment.

**Fig. 1:**
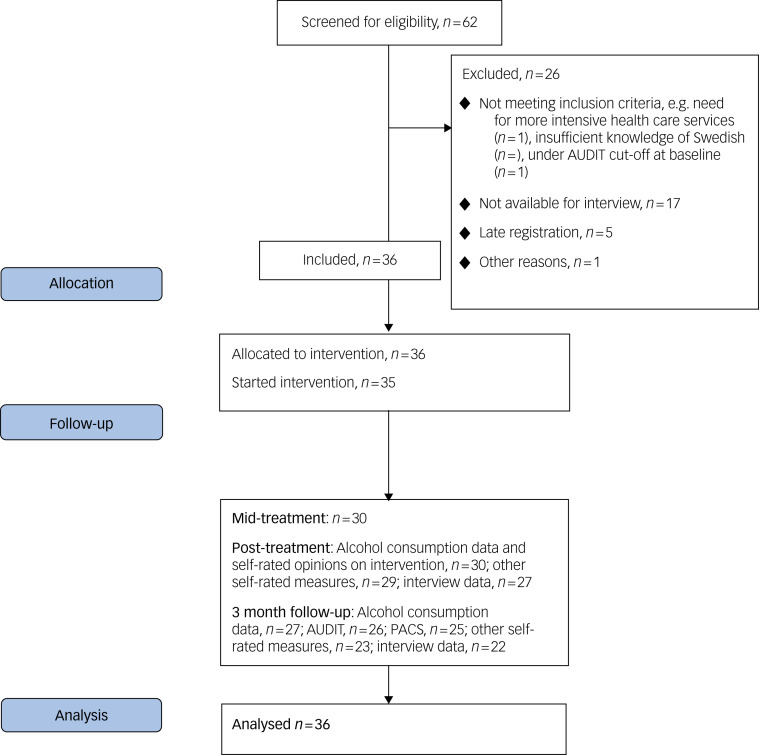
Study flow chart.

Prospective participants who filled out the survey were contacted for a telephone assessment, where suitability for DPSC was assessed as per inclusion and exclusion criteria. If included, presence of alcohol use disorder (AUD) was assessed by administering the AUD section of the Structured Clinical Interview for DSM-5 (SCID-5).^16^ Participants deemed to be in need of more intensive care were excluded and referred to adequate care. Those included were informed that they would receive access to the digital intervention on the following Monday. Upon first log in to the platform, participants filled out a pre-treatment assessment and were then given access to the digital intervention for 8 weeks, during which time no support was given, aside from technical support on request. Self-rated assessments were collected at pre-treatment, mid-treatment (after 4 weeks) and post-treatment (after 8 weeks) and at 3-month follow-up. A psychologist conducted follow-up interviews at post-treatment and again at the 3-month follow-up. In the post-treatment interview, the psychologist asked questions about how the intervention had been perceived. At the 3-month follow-up, a second assessment of AUD using SCID-5 was conducted. The study flow chart can be found in Fig. 1.

### Digital psychological self-care

In the current trial, we defined DPSC as a self-guided digital intervention focused on facilitating behaviour change provided within a structured care process that included clinical interviews conducted before and after the intervention. The telephone interviews before and after the intervention were hypothesised to serve as motivation for the participant to adhere to the intervention and to be a crucial intervention component for high levels of engagement with the main intervention components.

#### Digital intervention

The digital intervention consisted of techniques from cognitive–behavioural therapy (CBT) and relapse prevention (Table 1) and was based on an intervention used in an earlier randomised trial.^14^ For the current study, texts were considerably shortened and simplified to fit the self-guided, smartphone-friendly format.

**Table 1:** Content of the intervention and word length for mandatory and optional texts

Component	Description	Word count mandatory texts	Word count optional texts
Alcohol diary	Digital tool for goal setting and daily registration of standardised drinks. Goal fulfilment is calculated automatically and provided back to the user with along with a visual presentation.	302 words	939 words
Weekly module 1	Psychoeducation regarding alcohol and alcohol problems, reasons to change and goal setting. Introduction of patient narratives and instructions on how to use the alcohol diary. Summary of important strategies more comprehensively introduced in weeks 2–4.	4837 words	2836 words
Weekly module 2	Identifying risk situations and strategies for handling them.	1259 words	1128 words
Weekly module 3	Strategies for abstaining and for drinking moderately.	2970 words	1570 words
Weekly module 4	Strategies for scheduling alternative activities, maintaining progress and relapse prevention.	1753 words	845 words
Weekly module 5	Evaluation of progress and setting a new goal for the next 4 weeks.	841 words	506 words
Weekly module 6	Encouragement to continue using the alcohol diary and strategies.	99 words	351 words
Weekly module 7	Encouragement to continue using the alcohol diary and strategies.	99 words	299 words
Weekly module 8	Encouragement to continue using the alcohol diary and strategies.	99 words	384 words
Maintenance plan	Construction of a long term maintenance plan and advice on relapse prevention.	269 words	301 words

The alcohol diary guided participants to set goals for their alcohol consumption for 4 weeks at a time. There were three types of goals that participants could set:

- maximum number of standardised drinks (12 g of ethanol) per week;
- maximum number of standardised drinks during a single day;
- minimum number of alcohol-free days per week.

Every morning during the 8-week intervention, a text message (SMS) was sent to the participant with a prompt to log in and fill in the previous day's consumption in the alcohol diary. Together with the alcohol diary, there were weekly modules with instructions, strategies and examples. Aside from the main psychoeducational material and instructions, the following optional materials were available for the participants: answers to frequently asked questions based on the previous trial,^17^ four patient narratives and in-depth reading. Table 1 provides an overview of the intervention components and the word length of the different components. The total word length for the main material was 12 528 words. Optional in-depth reading, question-and-answer sections and example patient narratives added 9159 words, giving a total of 21 687 words. Most of the mandatory reading was located at the beginning of the 8-week intervention, with the second 4-week period acting as a bridge to managing one's alcohol consumption without being in a structured intervention. The intervention was designed around the use of the alcohol diary. The user interface of the alcohol diary and psychoeducational modules were made user-friendly, employing a mobile-first design approach with automated features and stepwise presentation in small chunks, as this may facilitate high levels of behavioural engagement.^18^ A screenshot of the user interface in the alcohol diary can be found in Supplementary Fig. 1 available at https://doi.org/10.1192/bjo.2023.73.

### Outcome measures

#### Intervention adherence

During the 8 weeks of the intervention, the participant could register a maximum of 56 days in the alcohol diary, indicating maximum adherence. In addition, at the post-treatment assessment, participants could rate how often they had used the intervention (including reading texts and using strategies), with four options: daily or almost daily, several times a week, around once a week, or never or almost never.

#### Credibility, user-friendliness and usefulness

Intervention credibility was measured at the post-treatment assessment with a five-item version of the Credibility/Expectancy Questionnaire.^19^ The scale consists of five 11-point items (0–10), yielding a score of 0–50 points in total, with a higher score reflecting higher treatment credibility. A total score of 30 points or higher is considered to indicate adequate treatment credibility

Perceived user-friendliness of the intervention was measured with the System Usability Scale.^20^ The scale consists of ten five-point items relating to usability, such as ‘I think that I would like to use this system frequently’, and scored (0–4) from Strongly disagree to Strongly agree. The item scores are summed up and calculated with a formula resulting in a total score between 0 and 100, with a higher score reflecting better system usability and 70 points or above considered to indicate acceptable usefulness.^21^

Usefulness of the different intervention components was rated at the post-treatment assessment using a study-specific question with five options: no or almost no usefulness, small usefulness, average usefulness, large usefulness, very large usefulness.

As for general perceptions, at the post-treatment assessment, participants responded to four study specific questions about whether (a) the intervention was likable, (b) the intervention was easy to understand, (c) the example patient narratives felt relevant, and (d) functionality and information contributed to the participant feeling overwhelmed. These four questions were answered on a four-point scale from 0 (strongly disagree) to 3 (strongly agree). Response alternatives for the ‘Overwhelmed’ item were reversed and scored from 3 (strongly disagree) to 0 (strongly agree), reflecting being overwhelmed as negative. A score above 1.5 could be said to indicate a positive attitude on all four items.

#### Clinician time

The time the psychologist spent on interviewing participants was logged as a measure of resource consumption.

#### Primary outcome (alcohol consumption)

At all assessment points, participants reported the number of standard drinks consumed during each of the preceding 7 days.^22^ This number was used to present the total number of drinks and to calculate the number of heavy drinking days (HDD) in the preceding week, with HDD defined as ≥5 (men) or ≥4 (women) standard drinks on a single day.^23^ At both follow-ups, the alcohol consumption data was also dichotomised into variables showing the number of participants at each assessment who (a) were abstinent (0 drinks), (b) consumed <10 drinks per week (proposed Swedish cut off applicable to all genders^24^), (c) had a >50% reduction since the pre-assessment, and (d) had no heavy drinking days. Low-risk drinking was defined as reporting <10 drinks in a week and having no heavy drinking days. In the case of missing self-rated alcohol consumption data at the post-intervention and 3-month follow-up time points, consumption was assessed during the telephone interview instead.^25^

#### Secondary outcomes

The following instruments were administered at screening and at the pre-intervention, post-intervention and 3-month follow-up assessments: AUDIT, Penn Alcohol Craving Scale (PACS^26^), Brunnsviken Brief Quality of Life Scale (BBQ^27^), nine-item Patient Health Questionnaire (PHQ-9^28^) and seven-item Generalized Anxiety Disorder questionnaire (GAD-7^29^).

#### Adverse effects

Participants were asked to report any adverse effects of the intervention during the mid- and post-treatment self-rated assessments.

### Statistical analysis

This paper presents the primary analysis of the described feasibility study. Analyses were conducted using SPSS Statistics 28 for Windows (IBM Corp.). Descriptive statistics are used to present observed data. Bias-corrected within-group effect sizes were calculated on observed data using Hedge's *g* and are presented with 95% confidence intervals. A power calculation suggested that 26 participants were needed for a power of 80% to detect a preliminary within-group effect size of *g* = 0.80, based on observed within-group changes in a previous study of two similar internet interventions.^14^ To allow for some attrition, the goal was to recruit around 30 participants. The aim was also to recruit around 30 participants to be able to provide sufficiently reliable feasibility estimates.

As an additional analysis, generalised estimating equations (GEE) with uncorrelated correlation structure and a negative binomial probability distribution were used to create estimated marginal means for alcohol consumption (drinks per week), based on all time points from the pre-treatment assessment to the 3-month follow-up, with the screening assessment used as a covariate.

## Results

### Participants and data attrition

The inclusion stopped when the desired number of participants was reached. Thirty-six participants were included in the study (Fig. 1). The majority of these were female, had a university degree, worked full-time and were not in a current relationship. Seventy-eight per cent had moderate or severe AUD according to the SCID interview, whereas only three of 36 participants (8%) had 0–1 AUD criteria and could therefore be classified as subclinical. Participant characteristics are presented in Table 2.

**Table 2:** Participant characteristics at screening (*n* = 36)

Variable	
Female, *n* (%)	21 (58%)
Age, mean (range)	50.0 (21–65)
Highest level of education, *n* (%)	
Elementary school	0 (0%)
Senior high school	14 (39%)
University	22 (61%)
Married or partner, *n* (%)	13 (36%)
Employment, *n* (%)	
Full or part-time	22 (61%)
On sick-leave or unemployed	9 (25%)
Retired or other	5 (14%)
Drinks preceding week, mean (s.d.)	20.8 (13.6)
Heavy drinking days preceding week, mean (s.d.)	2.39 (1.75)
Alcohol problem severity, AUDIT score, mean (s.d.)	18.4 (6.2)
Contact with addiction care services ever, *n* (%)	9 (25%)
Craving, PACS score, mean (s.d.)	14.9 (5.2)
DSM-5 Alcohol Use Disorder – subthreshold, *n* (%)	3 (8%)
DSM-5 Alcohol Use Disorder – mild, *n* (%)	5 (14%)
DSM-5 Alcohol Use Disorder – moderate, *n* (%)	14 (39%)
DSM-5 Alcohol Use Disorder – severe, *n* (%)	14 (39%)
Number of DSM-5 criteria, mean (s.d.)	5.0 (2.2)
Readiness to change questionnaire phase, *n* (%)	
Precontemplation phase	0 (0%)
Contemplation phase	33 (92%)
Action phase	3 (8%)
Quality of life, BBQ score, mean (s.d.)	56.9 (20.1)
Depression, PHQ-9 score, mean (s.d.)	7.3 (5.5)
Anxiety, GAD-7 score, mean (s.d.)	5.3 (5.6)

1, very poor; 5, very good; AUDIT, Alcohol Use Disorders Identification Test; PACS, Penn Alcohol Craving Scale; BBQ, Brunnsviken Brief Quality of Life Scale; PHQ-9, Patient Health Questionnaire (nine-item scale); GAD-7, Generalized Anxiety Disorder (seven-item scale).

Thirty participants (83%) provided drinking outcome data at the post-assessment follow-up, and 27 (75%) did so at the 3-month follow-up. Owing to some participants not finishing the whole follow-up assessment battery, and some participants being hard to reach by phone, some secondary outcome measures were responded to by 22–26 participants (61–72%). Data attrition is presented in Fig. 1.

### Intervention adherence

Engagement with the alcohol diary was high, with participants registering on average 42.5 of 56 diary entries (76%). One participant did not use the alcohol diary at all. Twenty-five of the 30 (83%) participants who completed the post treatment follow-up reported having used the intervention daily or several days per week, during the 8-week intervention period.

### Perceived usefulness and credibility

Most participants reported that they liked the intervention, that they found it easy to understand, that the example patient narratives were relevant and that they were not overwhelmed by it, with the average self-rated perceptions all being moderate to high on the positive side. Average usability and credibility ratings were above their respective cut-offs (Table 3). Please see the Supplementary material for more on perceived usefulness and participants’ comments.

**Table 3:** Number of diary entries, self-rated use, perceptions, usefulness and credibility of intervention, as well as clinician time spent per patient

Variable	*N* (%)	
Number of diary registrations (0–56), mean (s.d.)	36 (100%)	42.5 (17.1)
Self-rated use of intervention	30 (83%)	
Daily or almost daily, *n* (%)		17 (57%)
Several times a week, *n* (%)		8 (27%)
Around once a week, *n* (%)		3 (10%)
Never or almost never, *n* (%)		2 (7%)
Self-rated perceptions of intervention (scale 0–3)	30 (83%)	
Likable, mean (s.d.)		2.0 (0.6)
Easy to understand, mean (s.d.)		2.4 (0.7)
Relevant examples, mean (s.d.)		2.1 (0.6)
Feeling overwhelmed, mean (s.d.)^a^		1.9 (0.9)
System Usability Scale (0–100), mean (s.d.)	29 (81%)	80.3 (17.4)
CEQ (0–50), mean (s.d.)	29 (81%)	32.9 (11.2)
Clinician time per participant in minutes	36 (100%)	
Initial interview, mean (s.d.)		33.9 (9.1)
Post interview, mean (s.d.)		21.9 (17.4)
3 month follow-up interview, mean (s.d.)		11.2 (10.7)
Total clinician time, mean (s.d.)		67.0 (31.9)

CEQ, Credibility/Expectancy Questionnaire.

a. Reversed scoring, i.e. higher score indicates less overwhelmed

### Clinician time

The time spent on phone calls was just over 60 min per participant. The initial assessment interview was the most time-consuming part, taking more than 30 min per participant (Table 3). The average clinician time spent on the first two interviews, which were hypothesised to be an important part of the intervention, was slightly under 1 h.

### Primary outcome (alcohol consumption)

At follow-up, we found reductions in both drinks and HDD (Table 4). Effect sizes were moderate to large, with confidence intervals not including 0. The additional estimated means from the GEE model were similar to the observed means. An omnibus test of the GEE model for drinks per week was statistically significant (Wχ^2^ = 25.04, *P* < 0.001). Immediately after the intervention, 50% of participants were classified as low-risk drinkers, whereas at the 3-month follow-up, 41% were classified as low-risk drinkers. The number of completed alcohol diary entries was negatively correlated with the number of standard drinks per week post-intervention (*r* = −0.40, 95% CI = −0.67 to −0.05). Please see the Supplementary material for an exploratory comparison of low-risk and non-low-risk drinkers post-intervention.

**Table 4:** Alcohol consumption, means with standard deviations and confidence intervals, with both observed data and estimated means from the GEE model; within-group effect sizes and dichotomised outcomes were calculated compared with the pre-treatment time point

	Pre-treatment	Mid-treatment	Post-treatment	3-month FU
*N*	36	30	30	27
Drinks per week				
Observed score mean (s.d.)	22.6 (15.2)	12.2 (11.4)	10.6 (12.4)	12.7 (12.1)
95% CI Observed score	17.5-27.7	7.9-16.4	6.0-15.2	8.0-17.5
Estimated score mean (s.e.)	22.6 (2.49)	11.8 (1.93)	10.4 (2.11)	12.5 (1.99)
95% CI Estimated score	18.2-28.1	8.6-16.3	7.0-15.5	9.1-17.1
Effect size (Hedge's *g*)		*g* = 0.76	*g* = 0.85	*g* = 0.70
95% CI Effect size		0.26–1.26	0.34–1.35	0.19–1.21
0 drinks (abstinent) *n* (%)	2 (6%)	4 (13%)	7 (23%)	5 (19%)
<10 drinks per week *n* (%)	6 (17%)	13 (43%)	17 (57%)	13 (48%)
>50% reduction *n* (%)		12 (39%)	16 (53%)	9 (33%)
Heavy drinking days				
Observed score mean (s.d.)	2.53 (1.96)	1.47 (1.76)	1.30 (2.15)	1.37 (1.86)
95% CI Observed score	1.86-3.19	0.81-2.12	0.50-2.10	0.63-2.11
Effect size (Hedge's *g*)		*g* = 0.56	*g* = 0.59	*g* = 0.60
95% CI Effect size		0.07-1.05	0.10-1.09	0.09-1.11
0 HDD *n* (%)	4 (11%)	11 (37%)	18 (60%)	12 (44%)
Low-risk drinking *n* (%)	3 (8%)	9 (30%)	15 (50%)	11 (41%)

GEE, generalised estimating equation; FU, follow-up; HDD, heavy drinking days.

**Fig. 2:**
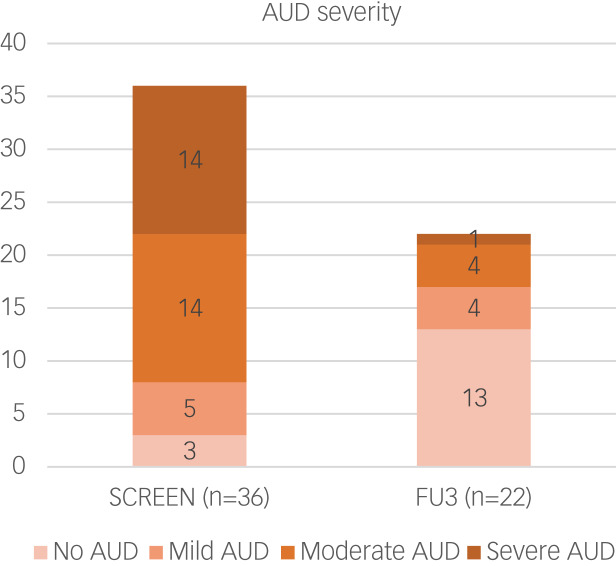
Levels of severity based on diagnostic interview. AUD, alcohol use disorder; SCREEN, initial assessment interview; FU3, follow-up interview 3 months after intervention.

We compared the number of AUD criteria in the SCID interview at 3-month follow-up with the number at the initial interview. Although this comparison was only based on 22 participants (61%), the average number of DSM-5 criteria was markedly lower (1.6, s.d. = 1.8) at the 3-month follow-up interview than at the initial interview (5.0, s.d. = 2.2). Of the 22 interviewed participants, 18 were at a lower AUD severity level compared with before the intervention, four were at the same level and none had changed to a more severe level. Please see Fig. 2 for levels of severity based on the number of AUD criteria.

### Secondary outcomes

Although there were indications of moderate to large within-group effect sizes on AUDIT and PACS at the 3-month follow-up, there were no consistent within-group effects on the quality of life scale (BBQ), the depression scale (PHQ-9) or the anxiety scale (GAD-7) (Table 5).

**Table 5:** Secondary outcomes. Observed values for alcohol problem severity, craving, quality of life, depressive severity and anxiety severity. Means (M), standard deviations and 95% confidence intervals at the available assessments; within-group effect sizes with 95% confidence intervals calculated compared with the pre-treatment time point

	Pre-treatment (*n* = 36)		Mid-treatment (*n* = 30)		Post-treatment (*n* = 29)		3-month follow-up (*n* = 26)		Within-group effect size from pre (Hedge's g) [CI]		
	M (s.d.)	CI	M (s.d.)	CI	M (s.d.)	CI	M (s.d.)	CI	Pre-mid	Pre-post	Pre-FU3
Alcohol problem severity^a^	17.4 (7.8)	14.8–20.0	–	–	13.2 (5.9)	10.9–15.4	10.8 (7.1)	7.9–13.6	–	0.59 [0.10–1.09]	0.87 [0.34–1.40]
Craving^b^	14.6 (6.1)	12.6–16.7	11.6 (6.4)	9.2–14.0	11.6 (6.2)	9.2–13.9	10.2 (7.0) *n* = 25	7.4–13.1	0.49 [−0.01–0.98]	0.50 [0.00–0.99]	0.67 [0.15–1.19]
Quality of life^c^	56.4 (18.5)	50.1–62.7	59.5 (19.8)	52.2–66.9	60.7 (22.8)	52.1–69.4	56.4 (24.6) *n* = 23	45.8–67.0	0.16 [−0.32–0.65]	0.21 [−0.28–0.70]	0.00 [−0.52–0.52]
Depression severity^d^	7.6 (6.5)	5.4–9.8	5.9 (5.8)	3.8–8.1	5.7 (6.1)	3.4–8.1	6.8 (6.7) *n* = 23	3.9–9.7	0.27 [−0.22–0.75]	0.29 [−0.20–0.79]	0.12 [−0.40–0.65]
Anxiety severity^e^	4.9 (5.6)	3.0–6.8	3.6 (4.2)	2.0–5.1	3.8 (5.4)	1.8–5.9	4.3 (5.6) *n* = 23	1.9–6.7	0.27 [−0.22–0.75]	0.20 [−0.29–0.69]	0.11 [−0.41–0.63]

a. Alcohol Use Disorders Identification Test.

b. Penn Alcohol Craving Scale.

c. Brunnsviken Brief Quality of Life Scale (higher is better).

d. Patient Health Questionnaire (nine items).

e. Generalized Anxiety Disorder questionnaire (seven items).

### Adverse effects

No adverse effects of using the intervention were reported by the participants.

## Discussion

### Main findings

The aims of this study were to evaluate the feasibility and preliminary effects of DPSC – that is, provision of an unguided digital intervention delivered within a structured care process – for problematic alcohol use. Seeing assessment and follow-up interviews as important components of the intervention is central to the concept of DPSC, in contrast to earlier research on guided and unguided digital interventions. In this study, almost 80% of participants had a moderate or severe AUD. As for feasibility, most participants reported logging in and using the alcohol diary frequently. Moreover, the digital intervention was deemed useful and credible, with no adverse events reported. As for preliminary effects, we observed a decrease in alcohol consumption with a moderate to large within-group effect size, which was maintained at the 3-month follow-up. At the 3-month follow-up, 41% were low-risk drinkers. Possible effectiveness regarding alcohol consumption needs to be examined in a larger controlled trial.

The high rate of use of the alcohol diary was encouraging. Registering in the diary on half the number of possible days during the intervention (28 days) or more could be considered as adequate adherence. Using that cut-off, 29 participants (81%) adhered to the alcohol diary adequately. This can be compared with the use of alcohol registrations in an earlier study, where the participants who received a digital self-help intervention without guidance only registered their alcohol use on average 3.8 times during a 10-week period, compared with 24.9 times for the group receiving written guidance.^13^ The clinician time spent on interviews of around 1 h per participant can be compared with the slightly less than 3 h clinician time per participant spent on written guidance in an earlier study of a more comprehensive and therapist-guided intervention based on the same CBT components.^14^ However, this previous study did not include time spent on clinical interviews, so the total clinician time may have been more than 3 h including both interviews and written guidance.

There was no decrease in alcohol consumption from screening to pre-intervention assessment for the participants included in the current study. Given the results of other recent randomised trials, this was surprising. In two trials of digital interventions by Sundström and colleagues,^14,30^ a decrease of around ten drinks from screening to pre-intervention assessment was noted. The less severe (on average) group in this study had a different trajectory, and possibly initial high consumption is predictive of a sudden pre-intervention decrease in consumption. The majority of the participants were well-adjusted individuals with full-time jobs, suggesting that we reached a sample of individuals who had not yet suffered great damage from their alcohol consumption. This could create a tendency to increase their consumption before the intervention start, as they knew it would have to decrease once they were in the intervention. This should be explored in future studies with larger samples. From the pre-assessment, however, alcohol consumption was very similar between the current study and the previous randomised trial,^14^ with the average drinks per week point estimates in the current study decreasing from 22.6 to 10.6 at post-intervention and 12.7 at follow-up, although with wide confidence intervals. By comparison, in the previously mentioned randomised trial, participants in the therapist-guided intervention decreased their alcohol consumption from 22.3 to 10.9 at post-intervention and 16.6 at 3-month follow-up, whereas those following the self-guided intervention decreased from 22.9 to 14.4 at post-intervention and 14.5 at 3-month follow-up.

Our results suggest that the absence of therapist guidance may be compensated by a user-friendly, engaging design and clinical interviews. This is in line with a recent large (*n* = 1169) randomised trial, where a self-guided digital intervention was compared with a therapist-guided intervention, and no significant differences in effects on alcohol consumption either directly after the intervention or at the 3-month follow-up were found.^31^ The proportion of participants classified as low-risk drinkers immediately after the intervention (50%) and at the three-month follow-up (41%) in the current study was also encouraging when compared to the larger study (43% at both corresponding time points).^31^ The authors of the previous study hypothesised that other forms of human contact, such as the use of online forums, may compensate for the lack of regular guidance. Another possible explanation for similar effects between self-guided and therapist-guided interventions is that patients’ need of a relationship with the therapist seem to vary, with self-guided interventions possibly being effective for already motivated clients.^32^

Secondary outcome measures of quality of life, depression and anxiety symptoms did not change much in this sample, a lack of effect that could possibly be explained by participants’ relatively high quality of life and low levels of depression and anxiety at baseline. The intervention needs to be examined in randomised trials with larger numbers of participants to draw any certain conclusions regarding effects on outcomes.

### Strengths and limitations

Strengths of this study include the diagnostic assessment interviews conducted with participants to assess severity of alcohol problems and the measurement of clinician time spent on interviews, which may be a crucial component for stimulating engagement with the intervention. Limitations include the non-controlled study design, rendering any conclusions regarding effects on outcomes preliminary. Another limitation is the data attrition, especially at the 3-month follow-up. Participants with missing data at the 3-month follow-up had characteristics and baseline alcohol consumption similar to those of the whole sample but showed relatively low adherence to the intervention, with a mean number of 31.1 diary entries compared with 42.5 in the whole sample. The measurements of clinician time spent on telephone interviews are potentially clinically useful, but as the interviews were a combination of clinical motivational efforts and research assessments it is hard to disentangle how much time and what interview content was needed to support the participants’ engagement with the intervention. To a large extent, the same psychologist did the assessment and follow-up interviews for a participant, as this was seen as a way to boost adherence to the intervention. This may have introduced bias in outcomes from the follow-up interviews. However, these outcomes were restricted to the question on preferred guidance and the participants’ subjective perceptions presented in the supplementary material. All other outcomes were self-rated online.

### Clinical implications

One important question is to what extent DPSC is more cost-effective than therapist-guided interventions, as both entail clinician time. Future studies could investigate DPSC in cost-effectiveness analyses. On average, 67 min of clinician time per participant was needed for interviews. Without the follow-up interview, which is not necessarily needed to boost engagement, it was less than 1 h. We hypothesise that the phone interviews are important for intervention adherence, but future studies should examine exactly how much and what type of guidance (e.g. automated versus clinician, text versus audio, synchronous versus asynchronous, scheduled versus on-demand) is necessary for high adherence and adequate decreases in alcohol consumption, given different thresholds of clinician time that the healthcare provider can provide. A contribution of the current study is to provide a baseline of the levels of adherence and within-group effects that can be expected for DPSC paired with around 1 h of clinician time per participant, given a help-seeking population. From the healthcare provider's perspective, clinician time can be a major cost and a limitation to implementation of structured interventions. Also important is the time spent on the intervention by the participant, which can be described as an indirect cost of the intervention. In this study, we reduced text length and streamlined the content into fewer core processes compared with earlier versions of digital interventions for alcohol problems.^14^ We found no indicators of the content being too lightweight for the study population. This may also act as a baseline for future alterations to length or intensity of intervention content. How intensive and demanding of the user the optimal alcohol self-help intervention should be may vary from person to person, and future studies should explore different levels of intensity and look for predictors of successful personalisation of treatment. As the majority of participants in this sample were female and had a university degree, similar to a sample recently recruited in Swedish primary care^33^, the sample may differ from AUD patients currently in addiction care services in important ways, such as the need for clinical guidance. If implemented, DPSC may be a way to reach new patients not currently receiving care, rather than a way to treat current patients. In addition, DPSC might be naturally suitable for people who are at risk of developing but have not yet developed AUD. In the current study, we might have reached a sample that matches this at-risk population.

In conclusion, DPSC was feasible and preliminarily moderately effective in reducing alcohol consumption among participants with problematic alcohol use, with around 1 h of clinician time spent on phone interviews. If proven to be effective and efficient in larger trials, DPSC may be a suitable first step in a stepped care model.

## Data Availability

Deidentified participant data will be made available upon reasonable request to the corresponding author, M.K.

## Supplementary material

For supplementary material accompanying this paper visit http://doi.org/10.1192/bjo.2023.73. click here to view supplementary material
