# Supplementary material for: Digital psychological self-care for problematic alcohol use: feasibility of a new clinical concept
Source: BJPsych Open. 2023 May 24;9(3):e91. doi: 10.1192/bjo.2023.73 (PMC10228278; doi:10.1192/bjo.2023.73)
Supplement: Supplementary file 1 [file S205647242300073Xsup001.docx]

**Supplementary material**

***Perceived usefulness and comments***

During the post-treatment assessment interview, participants were asked on their subjective perceptions of the intervention as well as which type of additional clinician support they hypothetically would have preferred: an extra telephone call or weekly written guidance.

The component of the intervention the participants (n=30) perceived as the most useful were the alcohol diary (number who rated usefulness large or very large), 22 (73%). After that, in descending order of perceived usefulness, were: the assessment telephone interview, 16 (53%); the psychoeducational texts, 13 (43%); strategies for risk situations, 11 (37%); the example patient narratives, 11 (37%); strategies for maintaining progress and relapse prevention, 10 (33%); strategies for scheduling alternative activities, 7 (23%); strategies for drinking moderately, 7 (23%); strategies for abstaining, 6 (20%).

Two example comments from the participants can serve as an illustration of typical positive and negative perceptions of using the intervention.

Positive example comment: *“It worked well, I think filled in almost every day, the next day. Also, the SMS-reminder. [...] It was well written if you are worried about others reading it.”*

Negative example comment *“In the beginning I did it in the morning, then it became afternoon, then it fell away. [...] in the beginning, I just filled in. I was curious how it would affect me, if the study could give me anything, but I have always been fully aware from the beginning how much I drink. It has not been helpful in any way unfortunately.”*

During the post-treatment follow-up interview (n=27) when asked what, if any, type of additional support the participant would have preferred (weekly written support or one extra phone call mid-treatment), the answers were split evenly. While 11 (41%) preferred written support (Example: “Then you can answer when you have the time. I don’t like to book appointments”), 13 (48%) preferred an extra phone call during the intervention (Example: “It puts more pressure on you with a phone call”). Three (11%) participants stated no need for additional support.

Fig. S1. Screenshot of the alcohol diary used in the intervention, exemplifying the goals of maximum drinks per day, maximum drinks per week and days without alcohol per week.


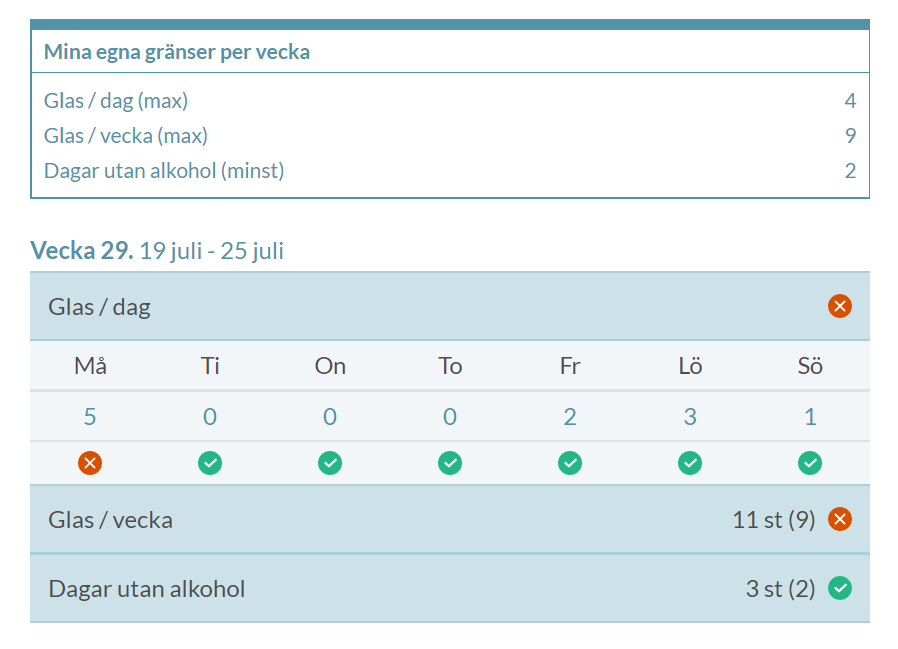


Table S1. An exploratory comparison of the participants who were/were not, low risk-drinkers at the post time point.

| Variable | Low risk-drinker at post (n=15) | Not low risk-drinker at post (n=15) |
| --- | --- | --- |
| Female, n (%) | 10 (67%) | 9 (60%) |
| Age, mean [95% CI] | 50 [43-57] | 51 [44-58] |
| Baseline drinks preceding week, mean [95% CI] | 15 [9-20] | 26 [17-35] |
| Baseline heavy drinking days preceding week, mean [95% CI] | 1.6 [0.9-2.3] | 3.4 [2.4-4.4] |
| Baseline alcohol problem severity, AUDIT score, mean [95% CI] | 18.5 [14.3-22.6] | 19.5 [16.6-22.4] |
| Number of diary registrations (0-56), mean [95% CI] | 52 [48-57] | 43 [35-50] |
